# Dietary Salt-Related Knowledge, Attitudes and Behaviors in Healthy and Hypertensive Turkish Adults from Food Choice Perspective

**DOI:** 10.3390/foods14010141

**Published:** 2025-01-06

**Authors:** Burcu Aksoy Canyolu, Beste Özben Sadıç

**Affiliations:** 1 Department of Nutrition and Dietetics, Faculty of Health Sciences, Istanbul Medeniyet University, Istanbul 34862, Turkey; 2 Department of Cardiology, School of Medicine, Marmara University, Istanbul 34854, Turkey; beste.ozben@marmara.edu.tr

**Keywords:** dietary salt, knowledge, attitude, behavior, food choice, Turkey

## Abstract

Food choices are influenced by knowledge, attitudes, and behaviors (KABs); therefore, determining KABs regarding salt is a key point for salt reduction, which is a primary public health problem in Turkey. This study aimed to assess salt-related KABs in Turkey. This cross-sectional study was conducted on 415 adults in a hospital in Turkey. A structured questionnaire assessing salt-related KABs was administered, and blood pressure was measured. The majority of participants were aware of the health risks associated with high salt intake. Higher overall attitude scores increased the odds of performing most behaviors to reduce salt, except for checking food labels, using spices instead of salt, and purchasing foods labeled as low-sodium, by almost three-fold. These results suggest that both normotensive and hypertensive adults need to improve their knowledge of the health effects and dietary sources of salt, and how to reduce it. Salt-related KABs were not found to be aligned with all positive attitudes toward salt consumption; this indicates the need for regulations that operate independently of public perceptions, such as policies for reducing salt in the food industry and making reduced-salt products more affordable to encourage healthier food choices.

## 1. Introduction

Food choice is a multifaceted issue with many dimensions related to products, consumers, and contexts, which have long been studied by researchers in various fields, especially food scientists [1,2]. Food choices are influenced by biological (e.g., hunger, appetite, and taste), socio-demographic (e.g., culture, family, and peers), psychological (e.g., mood, stress, and guilt), and economic (e.g., cost, income, and availability) factors, attitudes, values, and norms, as well as individuals’ knowledge and attitudes about food and nutrition [3,4,5]. Several studies that have been conducted in this have shown that more in-depth knowledge of certain foods and diets is linked to making healthier food choices that contain limited added sugar, saturated fat, and salt, and specific amounts of fruits, vegetables, dairy, and whole cereals [6,7,8,9]. In their study conducted with individuals from Portugal, Brazil, and Argentina, Ferrão et al. found that participants who had high knowledge of food perceptions were generally eating healthy [6]. It is clear that food choices have a large impact on individual and public health [1,4]. Some research related to this topic suggests that healthy food choices can be a long-term investment in future well-being, in addition to having a positive impact on current health status [10,11].

Conversely, food choices that are considered unhealthy or excessive consumption of certain foods might be associated with negative effects [12]. In this regard, understanding the individual motivations underlying food choice, especially food widely consumed in society or linked to some health problems, and determining attitudes and behaviors are key to changing consumption habits, shaping healthy behaviors, and protecting public health [12]. From this perspective, dietary salt has been used for centuries in Turkish cuisine (bread, pickles, soused foods, etc.), food preparation, storage, and flavoring processes. It has quite an important role in terms of taste [13].

Concomitant with this situation, salt consumption is quite high in Turkish society [14,15]. According to the results of “The Relationship between Hypertension and Salt Intake in Turkish Population (SALTURK-I and SALTURK-II)”, per capita, dietary salt consumption was determined as 18 g/day and 15 g/day, respectively [14,15]. The prevalence of diseases associated with increased salt consumption is also high in the Turkish population [16,17,18,19]. According to the “Changes in hypertension prevalence, awareness, treatment, and control rates in Turkey (The PatenT 2)”, 3 out of every 10 individuals in Turkey are hypertensive [16]. The prevalence of obesity is 33.2% in women and 18.2% in men [17]. The prevalence of lung, breast, colon–rectum, and prostate cancers in Turkey is 17.6%, 10.3%, 9.1%, and 8.3%, respectively [18]. Moreover, the prevalence of type 2 diabetes is 13.7% [19]. In accordance with the Global Burden of Disease Study (GBD 2016), it has been reported that 25.663 hypertensive individuals in Turkey died from ischemic heart disease and 12.971 died from cerebrovascular diseases [20]. The WHO recommends a reduction to <2 g/day in sodium (5 g/day salt) intake to reduce blood pressure and the risk of cardiovascular disease, stroke, and coronary heart disease in adults [19]. Moreover, the EFSA Panel on Nutrition, Novel Foods, and Food Allergens (NDA) considers that 2 g sodium/day is a safe and adequate intake for the general EU adult population [21]. In response to the increasing salt consumption around the world, the WHO aimed to reduce salt intake by 30% worldwide by 2025, in order to prevent and control non-communicable diseases and to stop the rise in diabetes and obesity rates [19]. In line with this goal, in Turkey, salt consumption awareness activities and various precautions have been implemented, and the Republic of Turkey Ministry of Health has pioneered Turkey’s Program for Reducing Excessive Salt Consumption (2017–2021) [22]. Within the scope of the action plan, the salt content of bread, olive, cheese, pastrami, and tomato paste has been reduced and various precautions such as not servicing salt at tables in restaurants and adding salt as necessary to the menus have been transferred into practice, in cooperation with the Republic of Turkey Ministry of Health and Republic of Turkey Ministry of Agriculture and Forestry [23]. Since 2015, The United Nations Development Program and the WHO have supported governments in developing national non-communicable disease (NCD) investment cases to identify the socioeconomic dimensions of NCDs [24]. In a study conducted within this scope reporting investment cases for NCDs in 13 countries (Armenia, Barbados, Cambodia, Ethiopia, Jamaica, Kyrgyzstan, Russian Federation, Philippines, Thailand, Turkey, Uganda, Uzbekistan, and Zambia) [25], it was determined that the public health intervention with the highest return on investment in the prevention and control of NCDs was reducing salt consumption [25]. It has been reported that a USD 1 million investment to reduce salt intake would save USD 88 million in health expenditure in Turkey over 15 years [25]. Within this scope, descriptive data on salt-related KABs and their relationship with socio-demographic predictors are a critical need to develop salt-related nutrition and health policies in Turkey [25]. Therefore, determining KABs regarding dietary salt is essential for understanding the individual motivations underlying salt consumption, creating permanent behavioral changes in individuals, protecting public health, and developing new action plans in the future [2]. Despite activities to reduce sodium/salt in processed packaged food and beverage products and to encourage a decrease in excessive salt consumption in the workplace within the scope of the action plan carried out in Turkey (2017–2021), hypertension is still one of the major public health problems [22,26], so determining KABs regarding dietary salt in individuals with hypertension will be an effective strategy for disease management, treatment, and cost reduction [16]. Various studies have been carried out in Turkey to determine salt consumption [14,15], evaluate the awareness of hypertensive individuals [27], and examine the relationship between anthropometric indices and blood pressure [28]. However, to the best of our knowledge, no study has yet been conducted to determine the KABs of Turkish adults regarding dietary salt consumption in healthy and hypertensive adults.

Strategies to modify salt intake include education aimed at changing societal norms regarding salt consumption at the public level, policy reforms and improved regulations, the design of food manufacturing techniques, and food labeling [29]. However, modifying the primary determinants of food and dietary choices, such as social and cultural factors and education level, can be challenging in the short term [30]. The salt intake of a population is believed to be influenced by the knowledge, attitudes, and behaviors that are regarded as convenient factors to adjust. WHO recommends the assessment of dietary salt-related knowledge, attitudes, and behaviors to initiate any intervention to raise consumer awareness [19]. The existing body of evidence from developed countries mostly presents Western and high-income populations and reports that salt- and sodium-related knowledge covering dietary sources, recommended daily intake, or actions to decrease consumption is still lacking [31]. The findings of the present study can theoretically identify the gaps that need to be focused on to improve the knowledge, attitudes, and behaviors of both healthy and hypertensive adults in Turkey before implementing actions designed to reduce the sodium intake of the population.

On the other hand, CVDs account for 47% of all deaths, just under a quarter of the population has hypertension, the average 15 g daily salt intake is three times higher than the recommendations of the WHO and FAO, and dietary salt-related KABs in Turkey have been under-represented [19]. Moreover, the Multisectoral Action Plan for Turkey Non-Communicable Diseases (2017–2025), which targets a 30% relative reduction in the mean population intake of salt/sodium and a 20% reduction in the prevalence of high blood pressure, will be completed very soon [32]. Acknowledging the assessment of dietary salt-related KABs is a key step in the development of effective interventions as recommended by the WHO. The results of this study could be a guide in developing or updating national salt reduction education and campaigns, and would also provide a foundation to stakeholders, policymakers, and the food industry for developing evidence-based salt reduction policies specific to Turkey. Because of its geographical location and deep-rooted history in providing health services, today, Turkey is also an important country in both health tourism and scientific developments in health sciences. The findings generated by this study would also provide information for neighboring countries to design further studies on dietary salt-related KABs to develop country-specific initiatives to reduce salt consumption. On the other hand, both the questionnaire and the results of the present study can be functional in the healthcare system, especially for dietitians, nutritionists, and physicians in their daily practice to examine the diet-related KABs of their patients or clients and develop personalized treatments, nutrition, and suggestions for hypertensive patients or normotensive individuals who consult them.

Accordingly, this study primarily aimed to determine KABs related to dietary salt among a sample of Turkish adults and to assess the differences by gender and blood pressure. Moreover, we aimed to determine the predictors of dietary salt-related behaviors.

## 2. Materials and Methods

### 2.1. Study Design and Participants

This cross-sectional study was conducted between November 2020 and September 2021. Non-hypertensive and old diagnosed hypertensive adults aged ≥18 years who applied to the cardiology outpatient clinic, healthcare professionals, and other hospital staff in a training and research hospital in Istanbul, Turkey, were included consecutively. Participants who had difficulties with communication, dementia, mental illness, and mental disability were excluded and newly observed hypertensive patients were not included. All participants gave written informed consent. A minimum sample size of 386 was required to achieve 95% power with a significance level (α) of 0.05. This was calculated using an online sample size calculator (https://select-statistics.co.uk/calculators/, accessed on 12 September 2020). Initially, 451 participants were included in the study. Then, 23 participants were excluded because they did not answer most of the questions, and 13 hypertensive participants could not complete their blood pressure measurements. Finally, the study was completed by 415 participants.

### 2.2. Measurements

#### 2.2.1. Research Instrument

A self-administered questionnaire that included multiple-choice closed-ended questions was used to assess KABs related to salt and sodium intakes. The questionnaire was modeled from previous related studies [33,34,35,36,37,38,39,40,41,42,43,44,45,46,47,48]. All stages are explained in detail below.

First, a comprehensive examination of the scientific literature related to KABs regarding salt was conducted using keywords (KABs, dietary salt, population, knowledge, attitude, behavior, etc.) on the Web of Science, PUBMED, and Google Scholar databases. Then, questions from previous related studies [33,34,35,36,37,38,39,40,41,42,43,44,45,46,47,48] were collected by researchers and examined by the expert team formed. The expert team included two nutritionists, two cardiologists, and one behavioral scientist, and was brought together by invitation through researchers’ professional and informal social networks. The attitude, behavior, and knowledge questions most frequently included in existing studies were selected to ensure consistency in the literature and compare research findings with data from different populations. A second elimination was made from the existing questions, taking into account Turkey’s food culture, traditional diet patterns, and eating habits. In particular, the results of SALTURK I and II [14,15], current campaigns to reduce salt consumption, and national food consumption research [49] were accepted as major criteria in the survey modification. Moreover, the clinical experiences of dietitians and cardiologists were also considered. Within this scope, according to the SALTURK I and II results [14,15], the answer to the question “How much salt do you think Turkish society eats?” has been determined. According to the culinary culture and the Turkey Nutrition and Health Research (TBSA) 2019 [49], the options for the question “Which of the following do you think is the main source of salt in the Turkish population diet?” were created. Moreover, considering the current salt reduction program, the question “Do you have any information about the ‘Reduction of Excessive Salt Consumption Program in Turkey’ of the Ministry of Health of the Republic of Turkey?” was included in the questionnaire [22]. Finally, the survey included four sections: demographic information, general information, views on dietary salt and salt intake, and thirty-three questions exploring knowledge of salt and sodium, consumer beliefs, and behavioral intentions related to salt reduction.

Content validity was assessed to test the extent to which the survey and questions served the purpose. For this purpose, in addition to the five researchers, five more researchers who were aware of the construct to be measured were reached out to. Ten experts were asked to provide their opinions on the questionnaire regarding its understandability, simplicity, and necessity. Expert opinion was then evaluated using the Content Validity Index (CVI). A CVI higher than 0.80 was considered acceptable for content validity [50], and the total CVI of this survey was 0.85. Subsequently, a pilot study was conducted with 50 individuals who had different characteristics (age, gender, and education status) selected from the study population to test the clarity of the questions. Comprehensibility of the items was examined through questions such as “What do you think this question asks?” or “What does this question mean?”. After the pilot study, some statements were simplified and completed. The questionnaire is presented as Supplementary Materials in this article (Supplementary File S1). Then, the validity and reliability of 14 multiple-choice knowledge questions regarding salt and salt consumption were evaluated using discriminative item analysis (index of difficulty and index of discrimination) and the Kuder–Richardson formula-20 (KR20). As a result, the knowledge questionnaire was valid and reliable, and all details are presented in Supplementary File S2.

The survey included four sections: demographic characteristics, general information, views on dietary salt and salt intake, and thirty-three questions exploring knowledge of salt and sodium, consumer beliefs, and behavioral intentions related to salt reduction.

Knowledge: The demographic characteristics assessed included age, sex, and education level. Participants reported the use of antihypertensive medication, household responsibility for grocery shopping, caring for a child under the age of 18, etc., in the general information section. Fourteen questions were used to assess the participants’ knowledge of dietary salt and sodium intake. The total knowledge scores were calculated based on the number of correct answers (responses to a test item were scored with 1 (correct) and 0 (incorrect)). This section included knowledge of the relationship between salt and sodium, dietary recommendations for salt intake, how population intake compares to the recommendation, dietary sources of salt in the Turkish population, the link between high salt intake and diseases, and whether there is any information about reducing excessive salt consumption program in the country.

Attitudes and behaviors: Six multiple-choice questions were used to assess attitudes and behaviors. Moreover, some common behaviors that can help reduce the salt consumption of participants were evaluated using questions with 3 options: ‘never do this’, ’sometimes do this’, and ‘often do this’.

This section included questions on the use of salt during cooking and at the table, whether a salt shaker was placed on the table, whether salt is added before tasting, and how often processed food products high in salt are consumed. In addition, there were questions on whether participants try to reduce the amount of salt and whether this is important for them.

#### 2.2.2. Blood Pressure Measurement

Blood pressure was measured in normotensive and old diagnosed hypertensive adults in order to relate it to KABs, not for diagnostic purposes. Measurements and diagnoses were made following the Turkish Endocrinology and Metabolism Association Hypertension Diagnosis and Treatment Guidelines (prepared in light of current global guidelines) [51,52].

Hypertension was defined based on systolic blood pressure (SBP) ≥ 140 mmHg and/or diastolic blood pressure (DBP) ≥ 90 mmHg and/or the use of antihypertensive medication [53]. Participants were advised not to smoke, drink alcohol, coffee, or tea, or exercise for at least 30 min, and individuals rested in a sitting position for at least 5 min before measuring blood pressure (BP). The arm to be measured was supported at heart level and the blood pressure cuff was placed on the bare arm, covering 80% of the arm circumference. Blood pressures were measured at the cardiology outpatient clinic of Marmara University Pendik Training and Research Hospital by an experienced nurse via an aneroid sphygmomanometer (215 004 02, Erka Perfect, Istanbul, Turkey) from the non-dominant arm while individuals were standing after 15 min of rest. Measurements were taken between 9:00 and 11:00 in the morning. Blood pressures were measured three times with an interval of 5 min and average BP was used to determine the individual’s blood pressure value.

### 2.3. Statistical Analysis

Statistical analysis was conducted using the Statistical Package for the Social Sciences version 23.0 (SPSS Inc., Chicago, IL, USA). Descriptive statistics were used to describe the independent variables (participants’ characteristics, knowledge, behaviors, and attitudes related to dietary salt). Sub-group analyses were based on gender (male, female) and blood pressure (normotensive, hypertensive). A range of Chi-squared (χ2) tests were carried out to compare groups (gender, blood pressure) across knowledge, attitudes, and behaviors regarding dietary salt and sodium. Independent variables affecting dietary salt-related behaviors were determined using binary logistic regression analysis. Odds ratios and 95% confidence intervals were reported using multiple logistic regression modeling. The significance level was determined to be <0.05. Dummy variables were created with the ‘create dummy variables’ command in the Statistical Package for the Social Sciences version 23.0 (SPSS Inc., Chicago, IL, USA) to add categorical variables to the model. A reference category was determined for each categorical variable, and dummy variables corresponding to this reference category were created. For example, in the ‘gender’ variable, ‘male’ was selected as the reference category and a dummy variable was created for ‘female’. Thus, the first category of each categorical variable was taken as the reference, and dummy variables corresponding to other categories were added to the model.

## 3. Results

### 3.1. Study Population

This study included 314 normotensive and 101 hypertensive individuals. The mean age of participants was 33.4 ± 13.7 years, and 61.0% of the sample were women. Moreover, 82.1% had a high school or higher education level. The mean systolic and diastolic blood pressures of normotensive participants were 113.1 ± 11.1 mmHg and 70.7 ± 8.3 mmHg, respectively, while those of hypertensive participants were 133.8 ± 13.3 mmHg and 86.5 ± 10.7 mmHg, respectively. The baseline characteristics of the study population are shown in Table 1. For blood pressure, the hypertensive group had significantly higher SBP and DBP (*p* < 0.01). In terms of education level, it was found that the education level was significantly lower in the hypertensive group (*p* < 0.01). The hypertensive group also had a significantly higher family history of hypertension (*p* < 0.05).

### 3.2. Knowledge Related to Salt Intake

Knowledge related to dietary salt was compared according to gender and blood pressure characteristics, and the results are shown in Table 2.

Overall, the vast majority of participants were aware that eating too much salt could damage health (95.1%). Similarly, most knew that eating too much salt was linked to hypertension and kidney diseases (93.4% and 89.9%, respectively). On the other hand, only 5.9% of the participants were aware that bread is the main source of salt in the typical Turkish diet and merely 19% of the study subjects knew that Himalayan salt, pink salt, sea salt, and gourmet salts are not healthier than regular iodized table salt. Less than half of the participants (37.7%) could correctly identify and were aware of the relationship between stomach cancer (36.5%), osteoporosis (33.6%), and consuming too much salt, and less than a third (27.1%) of participants had knowledge of about ‘The Excessive Salt Consumption Reduction Program in Turkey’. According to blood pressure sub-group analysis, a greater proportion of normotensive participants than hypertensive participants correctly identified the relationship between salt and sodium (60%, *p* < 0.001) and knew that consuming too much salt could cause hypertension, stroke, kidney diseases, and osteoporosis (95.8%, *p* < 0.005, 66.4%, 93.5%, *p* < 0.001, 37.9%, *p* < 0.05, respectively). Regarding salt-related knowledge across the gender sub-group, females could incorrectly identify the relation between Himalayan, pink, sea, and gourmet salt and health as well as the association between salt and kidney disease (*p* < 0.05).

### 3.3. Attitudes and Behaviors Related to Salt Intake

Attitudes and behaviors related to dietary salt were compared according to gender and blood pressure characteristics (Table 3). Almost half of the participants (45.6%) indicated that they usually placed salt shakers on their tables and most (69.0%) remarked that reducing their salt intake was not important to them. The overwhelming majority (93.7%) of individuals stated that they usually added salt before tasting or while eating. In the sub-group analysis, there were no differences in the declared salt-related attitudes and behaviors between males and females (*p* > 0.05), whereas a higher proportion of hypertensive participants minded about reducing their salt intake compared to normotensive participants (84.2%, *p* < 0.001), and salt addition behavior during cooking was significantly lower in the hypertensive group (89%, *p* < 0.05).

The most commonly reported behaviors to reduce salt intake among participants were avoiding eating fast food (38.5%) and eating at Asian cuisine restaurants high in salt (36.8%). Moreover, there was no significant difference between genders in terms of the frequency of behavioral practices. Conversely, hypertensive participants were more likely than normotensive participants to report avoiding eating at fast food restaurants and asking for meals prepared without salt when eating out (Table S1).

Salt-related behavioral practices were significantly associated with specific knowledge questions (Table S2). Those who knew that eating too much salt was linked to hypertension and kidney diseases were more likely to avoid processed food products with a high salt content. On the other hand, those who knew that eating too much salt was linked to stroke were more likely to add salt before tasting or while eating.

Salt-related behavioral practices were significantly associated with demographic features and blood pressure, as shown in Table 4. A significantly higher proportion of participants who had a secondary school education level or below and high blood pressure consumed processed food products with a high salt content. Moreover, significantly more men adopted favorable salt-related behavior, such as the purchase of foods labeled with no added salt.

Table 5 shows the association between salt-related behaviors and overall knowledge and attitude scores, socio-demographic characteristics, and blood pressure based on a multiple regression model.

The results showed that a higher overall attitude score increased the odds of performing all behaviors, except the three items (looking at a food label to check the salt/sodium content of a food item, using spices/herbs instead of salt when cooking, and purchasing foods labeled “no added salt”, “salt reduced”, or “reduced sodium”) related to reducing salt intake by almost three-fold. In other words, a higher attitude score was associated with higher odds of reducing salt intake. Similarly, with increased age, the odds of avoiding eating packaged ready-to-eat food (OR = 1.06; 95% CI: 1.002–1.121), avoiding eating food from fast food restaurants (OR = 1.10; 1.03–1.19), and when eating out, asking to have meals prepared without salt (OR = 1.07; 1.00–1.14) increased by almost two times. Furthermore, women were almost four-fold more likely than men to avoid eating packaged ready-to-eat foods (OR = 3.85; 95% CI: 1.07–13.82).

A higher education level was associated with higher odds of avoiding eating food from fast food restaurants. The probability of high school and university graduates or above avoiding eating food from fast food restaurants was 19.95 times (*p* = 0.014) and 11.874 times higher than that of primary and secondary school graduates, respectively (*p* = 0.025).

## 4. Discussion

Individuals’ knowledge, behavior, and attitudes about food are one of the main predictors of their food choices, and more in-depth food knowledge has been linked to healthier food choices and directly to better well-being [4,5,8]. In Turkish cuisine, foods that contain salt (bread, pickles, soused foods, etc.) are important, and salt plays a basic role in giving flavor to meals [11]. Accordingly, salt consumption in society (around 15–18 g/day) is quite high [13]. As a result, the prevalence of hypertension, which is directly related to excessive salt consumption, is also quite high (3 of every 10 individuals in Turkey are hypertensive), and it is a severe public health issue in Turkey [14]. In Turkey, reducing salt consumption has been identified as a primary public health intervention for the prevention and control of non-communicable diseases. In this regard, determining KABs associated with salt consumption is the primary step in determining the underlying reasons for individuals’ food choices, determining the current situation, and developing policies and action plans that can encourage permanent behavioral changes.

To our knowledge, this is the first study to determine KABs related to dietary salt among a sample of Turkish adults and to compare hypertensive and normotensive adults with KABs related to dietary salt. The results showed that (both hypertensive and normotensive) Turkish adults’ awareness of salt was rather low. They had little knowledge about the effects of salt on health (except hypertension and kidney diseases), basic salt sources, recommended daily salt limits, salt and sodium relationships, salt types, and current salt reduction programs. Our results suggest that most participants did not sufficiently adopt behavioral practices that help reduce their salt intake, and discretionary salt use is a fairly common behavior among our study population. Our findings demonstrate that high general attitude scores predicted almost all salt reduction-related behaviors. Moreover, this study identified older age, higher education level, and female gender as characteristics associated with several behaviors toward favorable salt reduction.

### 4.1. Knowledge About Dietary Salt and Consumption

The level of knowledge about dietary salt among Turkish adults was quite low, and this finding is generally consistent with most previous studies conducted in different populations with the same purpose [33,34,35,36,37,38,39,40,42,45,46,47,48].

When these results were examined in detail, almost the entire study population (95%) was aware of the harmful effects of excess salt on health, similar to the populations in Australia (90%), Italy (97%), Vanuatu (83%), Africa (85%), Nepal (86.3%), Montenegro (85.8%), India (80%), and Mozambique (83.1%) [33,34,35,40,42,45,48,54].

However, compared to the Urban Slums in India and Jordanian populations, a lower percentage of these participants were aware of the negative effects of consuming excess salt on health [43,44]. This is likely because 82.1% of our study population had a high school education level or higher, while 48.8% of the Indian sample consisted of illiterate participants and 53.4% of the Jordanian sample consisted of participants with primary school education or less [43,44].

Similarly to our results, previous studies conducted in Alabama, Florida, and China and a systematic review of twenty-two studies found that the majority of populations were aware that high salt intake is associated with high blood pressure (Turkey: 93.4%; Alabama and Florida: 91.1%; China: 80.3%) [37,38,55]. However, many participants in these studies, such as ours, were unaware of other health conditions (stroke, osteoporosis, and stomach cancer) that are potentially associated with high sodium intake [37,38,55].

Our findings emphasize the need to improve scientific understanding, consumer knowledge, and awareness of the negative consequences of excessive salt consumption. In contrast to the populations of China, Ireland, Beirut, and Australia (80.4%, 64.7%, 75.0%, and 22.6%, respectively) only 5.9% of participants in this study knew the main source of salt in the diet of the population [33,36,38,39]. Per capita bread consumption in Turkey is quite high (≥15 years, 180 g/day) [49], and this results in bread being the main source of salt in Turkey. This issue may explain why participants unconsciously consumed high amounts of salt due to their lack of knowledge, even though they stated that ‘reducing salt intake is important for me.’ There is a clear need to raise awareness of the current high salt intake seen across Turkish society, as well as what foods contribute salt to the diet [47]. It is critical that initiatives to reduce salt consumption include three components: the food industry, the state, and consumers. Hence, it is important to make food products with lower salt content accessible and available through government and food industry collaboration as well as educate consumers to choose products with lower salt content, reduce discretionary salt use, and develop the habit of reading food labels [56,57].

Our findings demonstrate that most participants (37.7%) were unsure of the maximal limit for salt intake, and this result is consistent with previous studies conducted in China (35.0%), Beirut (32.4%), Australia (28%), and Vanuatu (36%) and a systematic review of 13 studies (~30%) [36,38,39,48,55]. This is different from the results of studies conducted in Ireland (10%) and India (5%) [39,48]. Our findings demonstrate that 52% of the study population correctly identified the relationship between sodium and dietary salt. This result is consistent with a study conducted in Beirut (55.6%), but it is inconsistent with a study conducted in Australia (33%) [33,36].

This study’s findings have also shown that, similar to the Australian population [33], less than half of the participants (42.5%) knew that there was no health difference between regular table salt and gourmet salt. This finding may indicate that consumers make mistakes when making food choices and have insufficient knowledge about reading food labels. Moreover, since 1994, table salt has been enriched with iodine as a nutritional policy in Turkey [58]. In this regard, preferring salts such as rock salt or Himalayan salt instead of table salt may negatively affect the fight against iodine deficiency.

Therefore, it is important to increase consumer awareness of the interpretation, food fraud, and understanding of food labels. Both food and nutrition literacy play an important role in protecting population health and allowing them to make healthy food choices [59]. Training on reading food labels and increasing understanding of labels can increase individuals’ motivation to make healthy food choices [59]. The results of this study show that although the “reducing salt consumption” program has been carried out in Turkey since 2017, less than one-third of the sample (27.1%) knew the subject [19].

A previous study conducted in Ireland found awareness to be much higher than in our study (88.2%) [39]. This result shows that current programs and plans are not sufficiently known by Turkish society. Active use of media channels and assignment of trainers, health workers, and authorities in various government, health, and educational institutions, specifically for this subject, may be recommended to increase awareness.

According to a study evaluating 96 national salt reduction initiatives conducted up to 2019 (40 in Europe, 19 in the Western Pacific, 18 in the Americas, 13 in the Eastern Mediterranean, 5 in Southeast Asia, and 1 in Africa), the most common interventions were intervention in settings (77%), food reformulation (71%), consumer education (52%), front-of-pack labeling (50%), and salt taxation (5%), and the greatest progress with these interventions has been made in high-income countries [31]. However, no country has yet reached the targeted 30% relative reduction in average salt intake compared to baseline or the recommended daily limit of 5 g/day [31]. Additionally, in 2016, the WHO published the SHAKE Technical Package for Salt Reduction to further support Member States in implementing their salt reduction strategies: surveillance, mobilizing industry, adopting labeling standards, information, and environment [60]. As a result, considering both WHO recommendations and interventions implemented in other countries, it is essential to continuously monitor and evaluate salt reduction initiatives in Turkey, revise them, and identify areas requiring support.

### 4.2. Salt Reduction Attitudes and Behaviors

Similarly to previous studies (China 65.6%, Australia 40.0%, Africa 91%, and Nepal 88.7%), more than half of the sample (69%) thought salt reduction was important [33,38,40,42], and 50.2% of the participants stated that they had tried to reduce the amount of salt they consumed. However, the frequency of behaviors aimed at reducing salt consumption among participants was quite low, and discretionary salt use was quite high.

These research findings showed that almost the entire population (93.7%) stated that they ‘often’ add salt to food during cooking. These findings were in agreement with those reported for samples from various populations: Australia (38%), Africa (92%), India (85%), Jordan (80%), and Montenegro (74%) [33,35,43,44,45]. And similarly, almost half of the participants (45.6%) stated that they ‘often’ put salt on the table. These results support the finding that salt consumption is high among Turkish adults [12,13]. This may explain why ‘thinking that reducing salt is important’ is not a strong enough motivation for changing behavior in individuals. Moreover, considering that only 27.1% of the participants knew the Reducing Excessive Salt Consumption (2017–2021) program carried out between 2017 and 2021, it can be said that despite many actions being taken, sufficient awareness has not been created in society [22].

Within this scope, there is a need for campaigns and messages that motivate individuals to change their behavior. It is important to develop and revise campaigns and messages according to the WHO strategies (surveillance, mobilizing the industry, adopting labeling standards, information, and the environment [60]. Moreover, food reformulation, consumer education, front-of-pack labeling, and salt taxation interventions that have been successful in other countries may be effective, and they should also be evaluated, updated, developed, or implemented with the cooperation of the government, scientists, and food institutes. Consumption of Asian foods and fast food is not widespread, especially among middle-aged and older Turkish adults (the study simple age mean: 33.4 ± 13.7 years). Asian foods are costly and accessibility is limited in Turkey [61]. Additionally, consistent with previous studies conducted in Alabama and Florida, this study determined that only a small percentage of participants were checking for sodium content in food products (8.4%) and always buying low-sodium products (15.5%) [37].

Our findings emphasize that reading food labels is not yet widespread among consumers, and their importance for their health or well-being is not understood. In terms of food choice motivation, it can be concluded that the high sodium content of food is not an important source of food choice motivation for Turkish adults. It is clear that marketing and pricing policies should be developed in order to make a habit of reading food labels to increase the consumption of products with low sodium content and increase their accessibility and availability. Therefore, it would be beneficial to highlight consumer education and front-of-pack labeling strategies, which have been reported to be effective interventions, especially in developed countries, and to consider these strategies as important public health investments in the prevention of NCDs.

In this study, we investigated knowledge, attitude, and socio-demographic factors as predictors of salt-related behavior. The results obtained in this study identified higher overall attitude scores, older age, higher education level, and female gender as characteristics associated with favorable behaviors for salt reduction. Higher overall attitude scores were found to be significantly associated with almost all behaviors for reducing salt. More specifically, older age was found to be significantly associated with ‘avoiding eating packaged ready-to-eat food’, ‘avoiding eating food at fast food restaurants’, and ‘when eating out, asking to have meals prepared without salt’. These findings are in agreement with those of several previous studies that have shown that attitudes and older age are associated with favorable salt-related behavioral practices [36,56,62].

The findings of our study showed that both normotensive and hypertensive individuals in this sample had insufficient knowledge about salt, salt consumption, and related campaigns and, accordingly, did not adopt behaviors to reduce salt consumption. There is a need for effective strategic interventions and actions that can create behavioral changes in addition to increasing knowledge. It is important to develop and revise campaigns and messages according to the WHO strategies (surveillance, mobilizing the industry, adopting labeling standards, information, and the environment). Additionally, including all media channels in salt reduction initiatives can be advantageous, particularly in terms of educating consumers, raising awareness, and making messages more easily accessible. In particular, for hypertensive individuals, adding nutritional counseling by dietitians to routine follow-up after diagnosis so that they can follow their diets will be beneficial in increasing awareness of hypertension, providing behavioral changes, and indirectly improving health status.

This study had strengths and some limitations. First, to the best of our knowledge, this is the first study to evaluate dietary salt-related KABs and differences according to gender and blood pressure. The effects of various factors on salt reduction-related behaviors were evaluated using advanced statistical analysis. Finally, data were collected by expert researchers through face-to-face interviews and higher-reliability web-based studies. Besides these, this study has a few limitations. First, bias risks may have been introduced, such as self-selection bias and participants’ willingness to respond accurately and honestly, because the data were collected based on individuals’ declarations. Second, BP was measured in a single visit, and white coat syndrome may have caused the blood pressures of some participants to read higher than normal. Third, this study was conducted in a single center, so these results cannot be generalized to the whole country. Additionally, since the mean age of our population was 33.4 ± 13.7 years and 82.1% of the participants had a high school education level or higher, the results cannot be generalized to older individuals, especially those with low education levels and those who are illiterate. While this study provides a good overview of KABs, further qualitative research (e.g., focus groups or interviews) could complement the quantitative findings and provide deeper insights into participants’ beliefs and motivations regarding salt consumption.

Considering these limitations, further studies should be conducted with more diverse and representative samples to provide more generalizable results, especially with older adults and adolescents, who are a significant segment of consumers. It is accepted that the 24 h urinary excretion of sodium is an indirect measure of salt-related behavior; therefore, we recommend conducting advanced studies that use objective dietary assessment methods such as 24 h urinary excretion in the future. Furthermore, we recommend that studies be conducted to determine salt-related KABs in individuals with obesity, type 2 diabetes, and lung, breast, colorectal, and prostate cancers, which are associated with excessive salt consumption. In addition, longitudinal studies are recommended to track changes in KABs over time, following public health interventions.

## 5. Conclusions

Nutritional knowledge influences food choice and consumption. In the present study, it is obvious that both normotensive and hypertensive Turkish adults require education about the relationship between sodium and salt intake and its effects on health. Their knowledge and awareness should be increased, and behavioral changes should be provided.

The present study can theoretically contribute to existing literature with the results. Only 5.9% of participants identified bread as the primary source of salt in the typical Turkish diet; this might be because they were not fully aware of hidden sources of salt, especially for foods without a salty taste [63]. The findings of the present study suggest that there is a need to focus on low levels of knowledge of the salt content of foods, salt recommendations for adults, dietary salt sources, the link between sodium and salt in hypertensive adults, and the importance of iodized salt as a healthy choice in terms of salt consumption in the Turkish population since the table salt iodization program was launched officially in Turkey in 1998, which suggested that more than a decade of iodine prophylaxis would be needed to eradicate goiters in a moderately iodine-deficient region such as Turkey [63].

Even though it has been reported that women have greater nutrition knowledge and interest in healthy eating [64], in contrast, our results suggest that Turkish women have low knowledge of iodized salt consumption as a risk for iodine deficiency in Turkey, suggesting that Turkish women adopt the favorable behavior of purchasing foods labeled as no added salt less compared to men, while men are almost four times less likely than women to avoid eating packaged ready-to-eat foods.

These findings indicate that there may be small but important differences in KABs regarding dietary salt among genders. Men and women need to be addressed separately to improve these gaps. Knowledge regarding the relationship between sodium and salt also needs to be addressed to encourage hypertensive adults, considering that 84.2% of hypertensive adults indicated that they were encouraged to reduce salt. The behavior of favorably adapting to avoid consuming processed food products was low in the hypertensive participants with secondary and below education level, and low-educational hypertensive adults need to be supported to improve their processed food consumption.

The present study revealed that a higher overall attitude score increased the odds of performing all behaviors, except for checking food labels for the salt/sodium content of food, using spices/herbs instead of salt when cooking, and purchasing foods labeled “no added salt”, “salt reduced”, or “reduced sodium”, which are related to reducing salt intake by almost three-fold. These behaviors should be taken into account in future efforts to target behavioral changes in terms of salt reduction. It has been shown that interest in health and nutrition increases with age [65].

This could be a result of the increased risk of NCDs with age, and a similar trend was observed in our study. The results showed that increased odds of avoiding eating packaged ready-to-eat food, avoiding eating food from fast food restaurants, and when eating out, asking to have meals prepared without salt were related to older age.

Reducing salt consumption was identified as a primary public health intervention for the prevention and control of non-communicable diseases in Turkey. In this direction, we foresee that the results of this study will significantly contribute to the development of salt reduction policies, which are considered important public health investments in the prevention of NCDs. In particular, we believe that older age, attitudes, and higher education level factors, identified as predictors of behaviors associated with reducing salt consumption, will provide an opportunity to design effective behavioral change interventions. Moreover, the study findings will be useful for healthcare professionals to examine the dietary-related KABs of their patients or clients to develop personalized treatments, nutrition plans, and suggestions for hypertensive patients or normotensive individuals who consult them, particularly about hypertension. In addition, our findings will guide the development of new national action plans, education programs, and awareness campaigns and the re-evaluation of the effectiveness of those currently implemented, focusing on the points identified as deficient and encouraging permanent behavioral changes under the WHO’s 2025 Global Action Plan. Furthermore, this study forms the basis for future national studies. For this purpose, we recommend that future national studies be conducted to include more confounding factors and evaluate causal relationships using advanced statistics.

The present study can practically contribute to existing literature with the results. The present study focused on the gaps in dietary salt-related KABs in normotensive and hypertensive Turkish adults, providing strong evidence regarding the need for interventions in terms of nutrition and health education for the population on salt intake and related health outcomes, the development of effective health and food policies, and action plans to bring behavioral changes and improve food choices around salt reduction. Policies and action plans would provide healthy options to populations; however, individuals still have the responsibility to prefer healthy choices in terms of their salt and sodium intake. The “Programme for reducing high salt consumption in Turkey” was conducted between 2017 and 2021 to prevent and control NCDs in Turkey. In response to this program, the food industry reduced the salt content of bread, tomato paste, and other processed foods, but according to the results of the “Turkey Household Health Survey (Prevalence of Risk Factors of Non-Communicable Diseases)” conducted by the Ministry of Health in cooperation with the WHO in 2017, daily salt consumption was found to be 9.9 g/day [23]. Despite the actions of policymakers and the food industry, the current high prevalence and burden of CVDs and hypertension in Turkey and the remaining high intake of dietary salt point out the need to assess and understand dietary salt-related KABs in Turkey, which is crucial for both the prevention and control of NCDs in Turkey.

The research results clearly show that both normotensive and hypertensive Turkish adults should be educated about the relationship between sodium and salt, salt intake, and its effects on health; their knowledge and awareness should be increased, and behavioral changes should be encouraged.

Further studies should be conducted on dietary salt-related KABs, food choices, and other related diseases, such as kidney disease, stomach cancer, and osteoporosis. In studies investigating KABs related to dietary salt, adult consumers were the target study population, as one of the criteria for most studies was only to include decision-makers directly involved in food purchasing and food choices; further studies are suggested to examine the dietary salt-related KABs of adolescents to determine the required actions around salt intake in the future. As a specific target group, the elderly population can also be investigated.

Finally, we recommend further studies to directly evaluate the impact of salt-related food preferences on current health status and salt-related health problems. In conclusion, encouraging consumers’ healthy food choices regarding salt consumption will undoubtedly help control NCD risks and promote health. The results of the present study are useful for understanding the KABs related to dietary salt and food choices in Turkey in both normotensive and hypertensive adults. Our results highlight a gap in understanding which KABs related to dietary salt topics and areas need to be highlighted to encourage people to adopt them for healthier food choices regarding salt and provide information to policymakers and the food industry to design required country-specific regulations, action plans, education, and other initiatives.

## Figures and Tables

**Table 1:** Sample characteristics in relation to blood pressure.

Characteristics	Total (n = 415)		Normotensive (n = 314)		Hypertensive (n = 101)		
	Mean ± SD	Min–Max	Mean ± SD	Min–Max	Mean ± Sd	Min–Max	*p*
**Age**	33.4 ± 13.7	18–88	30.2 ± 11.1	18–87	43.7 ± 16.0	18–88	<0.01
**Systolic blood pressure**	118.9 ± 15.0	79–161	113.1 ± 11.1	79–138	133.8 ± 13.3	98–161	<0.01
**Diastolic blood pressure**	75.2 ± 11.5	49–104	70.7 ± 8.3	49–90	86.5 ± 10.7	55–104	<0.01
	**n**	** %**	**n**	**%**	**n**	**%**	***p***
**Education level**							
Secondary school and below	74	17.8	29	9.2	45	44.6	<0.01
High school	186	44.8	164	52.2	22	21.8	
University and postgraduation	155	37.3	121	38.5	34	33.7	
**Gender**							
Male	162	39.0	104	33.1	58	57.4	>0.05
Female	253	61.0	210	66.9	43	42.6	
**Family history**							
Yes	202	48.7	141	55.0	61	61.0	<0.05
No	211	50.8	172	45.0	39	39.0	

Association assessed by Pearson’s Chi-squared test.

**Table 2:** Knowledge related to dietary salt by gender and blood pressure characteristics.

Knowledge Questions About Dietary Salt	Total		Normotensive		Hypertensive			Male		Female		
	n	%	n	%	n	%	*p*	n	%	n	%	*p*
**High salt consumption may damage health**												
True	392	95.1	297	95.5	95	94.1	>0.05	149	93.1	243	9.0	>0.05
False	20	4.9	14	4.5	6	5.9		11	6.9	96	3.6	
**Relationship between salt and sodium**												
True	211	52.4	183	60.0	28	28.6	<0.001	84	53.5	127	51.8	>0.05
False	66	16.4	50	16.4	16	16.3		20	12.7	46	18.8	
Don’t know	126	31.3	72	23.6	54	55.1		53	33.8	72	29.4	
**Salt consumption in Turkish adults *(15 g)***												
True	240	58.3	185	59.5	55	54.5	<0.001	88	55.0	151	60.2	>0.05
False	138	33.5	110	35.4	28	27.7		53	33.1	85	33.9	
Don’t know	34	8.3	16	5.1	18	17.8		19	11.9	15	6.0	
**Main source of salt in the diet of the Turkish population *(bread)***												
True	24	5.9	16	5.2	8	8.1	>0.05	13	8.1	11	4.5	>0.05
False	384	94.1	293	94.8	91	91.9		147	91.9	236	95.5	
**Himalayan salt, pink salt, sea salt, and gourmet salts are healthier than regular table salt**												
True	77	19.0	57	18.6	20	20.4	>0.05	34	21.4	43	17.6	<0.05
False	172	42.5	130	42.3	42	42.9		70	44.0	85	34.7	
Don’t know	156	38.5	120	39.1	36	36.7		55	34.6	117	47.8	
**Recommended daily salt intake not to exceed for adults *(5 g/day)***												
True	153	37.7	120	39.0	33	33.7	>0.05	67	41.9	85	34.7	>0.05
False	156	38.4	117	38.0	39	39.8		55	34.4	101	41.2	
Don’t know	97	23.9	71	23.1	26	26.5		38	23.8	59	24.1	
**Ministry of Health of the Republic of Turkey has a program called “Reduction of Excessive Salt Consumption Program in Turkey”**												
True	112	27.1	85	27.2	27	26.7	>0.05	41	25.5	71	28.2	>0.05
False	172	41.5	134	42.8	38	37.6		76	47.2	96	38.1	
Don’t know	130	31.4	94	30	36	35.6		44	27.3	85	33.7	
**High salt intake is associated with increased risk of hypertension**												
True	383	93.4	297	95.8	86	86.0	<0.005	147	91.9	235	94.4	>0.05
False	15	3.7	7	2.3	8	8.0		5	3.1	10	4.0	
Don’t know	12	2.9	6	1.9	6	6.0		8	5.0	4	1.6	
**High salt intake is associated with increased risk of stroke**												
True	245	61.6	200	66.4	45	46.4	<0.001	22	14.1	33	13.7	>0.05
False	55	13.8	37	12.3	18	18.6		91	58.3	153	63.5	
Don’t know	98	24.6	64	21.3	34	35.1		43	27.6	55	22.8	
**High salt intake is associated with increased risk of kidney disease**												
True	365	89.9	288	93.5	77	78.6	<0.001	133	84.7	231	93.1	<0.05
False	20	4.9	10	3.2	10	10.2		10	5.4	10	4.1	
Don’t know	21	5.2	10	3.2	11	11.2		14	8.9	7	2.8	
**High salt intake is associated with increased risk of heart attack**												
True	310	77.3	243	79.9	67	69.1	>0.05	14	9.0	20	8.2	>0.05
False	34	8.5	24	7.9	10	10.3		113	72.9	196	80.0	
Don’t know	57	14.2	37	12.2	20	20.6		28	18.1	29	11.8	
**High salt intake is associated with increased risk of stomach cancer**												
True	144	36.5	119	39.7	25	26.3	>0.05	36	23.4	49	20.4	>0.05
False	85	21.5	63	21.0	22	23.2		54	35.0	89	37.1	
Don’t know	166	42.0	118	39.3	48	50.5		64	41.6	102	42.5	
**High salt intake is associated with increased risk of osteoporosis**												
True	133	33.6	114	37.9	19	20.0	<0.05	45	29.2	88	36.5	>0.05
False	75	18.9	55	18.3	20	21.1		33	21.4	42	17.4	
Don’t know	188	47.5	132	43.9	56	58.9		76	49.4	111	46.1	

Association assessed by Pearson’s Chi-squared test. Correct answers are provided in brackets.

**Table 3:** Attitudes and behaviors related to dietary salt by gender and blood pressure characteristics.

Attitudes and Behaviors Related to Dietary Salt	Total		Normotensive		Hypertensive			Male		Female		
	n	%	n	%	n	%	*p*	n	%	n	%	*p*
**Place salt shakers on table**												
Usually	188	45.6	143	46.0	45	44.6	>0.05	72	45.0	116	46.2	>0.05
Sometimes	168	40.8	127	40.8	41	40.6		68	42.5	99	39.4	
Never	56	13.6	41	13.2	15	14.8		20	12.5	36	14.4	
**Add salt before tasting or while eating**												
Usually	79	19.1	56	18.0	23	22.8	>0.05	35	21.9	44	17.5	>0.05
Sometimes	189	45.9	147	47.3	42	41.6		69	43.1	119	47.4	
Never	144	35.0	108	34.7	36	35.6		56	35.0	88	35.1	
**Add salt during cooking**												
Usually	386	93.7	297	95.2	89	89.0	<0.05	139	87.4	246	97.6	<0.001
Sometimes	26	6.3	15	4.8	11	11.0		20	12.6	6	2.4	
**Consume processed food products with a high salt content**												
Usually	85	20.6	61	19.6	24	24.0	>0.05	36	22.5	49	19.5	>0.05
Sometimes	278	67.5	219	70.1	59	59.0		104	65.0	173	68.9	
Never	49	11.9	32	10.3	17	17.0		20	12.5	29	11.6	
**Try to reduce salt consumption**												
No	146	35.6	121	39.2	25	24.8	>0.05	54	34.0	92	36.8	>0.05
Yes	206	50.2	141	45.6	65	64.4		84	52.8	121	48.4	
Don’t know	58	14.1	47	15.2	11	10.8		21	13.2	37	14.8	
**Reducing salt intake is important to me**												
Yes	285	69.0	200	64.1	85	84.2	<0.001	106	66.2	178	70.6	>0.05
No	88	21.3	78	25.0	10	9.9		39	24.4	49	19.5	
Don’t know	40	9.7	34	10.9	6	5.9		15	9.4	25	9.9	

Association assessed by Pearson’s Chi-squared test.

**Table 4:** Association of salt-related behaviors with demographic features and blood pressure.

Variables	Place Salt Shakers on Table					Add Salt Before Tasting or While Eating					Consume Processed Food Products with High Salt Content					Check Food Labels for the Salt/Sodium Content					Purchase Foods Labeled “No Added Salt”, “Salt Reduced”, or “Reduced Sodium”					Try to Reduce Salt Consumption				
	Usually		Never		*p*	Usually		Never		*p*	Usually		Never		*p*	Usually		Never		*p*	Usually		Never		*p*	Yes		No		*p*
	n	%	n	%		n	%	n	%		n	%	n	%		n	%	n	%		n	%	n	%		n	%	n	%	
**Gender**																														
Male	140	87.5	20	12.5	>0.05	104	65.0	56	35.0	>0.05	140	87.5	20	12.5	>0.05	117	80.7	28	19.3	>0.05	97	80.8	23	19.2	<0.05	138	86.8	21	13.2	>0.05
Female	215	85.7	36	14.3		163	65.0	88	35.1		222	88.4	29	11.6		166	77.9	47	22.1		136	68.7	62	31.3		213	85.2	37	14.8	
Total	355	86.4	56	13.6		267	65.0	144	35.0		362	88.1	49	11.9		283	79.1	75	20.9		233	73.3	85	26.7		351	85.8	58	14.2	
**Education level**																														
Secondary school and below	62	84.9	11	15.1	>0.05	49	68.1	23	31.9	>0.05	56	77.8	16	22.2	<0.05	50	82.0	11	18.0	>0.05	31	70.5	13	29.5	>0.05	67	91.8	6	8.2	>0.05
High school	158	85.4	27	14.6		119	64.3	66	35.7		169	91.4	16	8.6		129	76.8	39	23.2		120	77.4	35	22.6		155	84.2	29	15.8	
University and postgraduation	136	88.3	18	11.7		100	64.5	55	35.5		138	89.0	17	11.0		105	80.8	25	19.2		83	69.2	37	30.8		130	85.0	23	15.0	
Total	356	86.4	56	13.6		268	65.0	144	35.0		363	88.1	49	11.9		284	79.1	75	20.9		234	73.4	85	26.6		352	85.9	58	14.1	
**Blood pressure**																														
Normotensive	219	86.6	34	13.4	>0.05	166	65.6	87	34.4	>0.05	232	91.3	22	8.7	<0.05	173	76.9	52	23.1	>0.05	150	72.8	56	27.2	>0.05	208	82.9	43	17.1	>0.05
Hypertensive	86	85.1	15	14.9		65	64.4	36	35.6		83	83.0	17	17.0		70	83.3	14	16.7		48	70.6	20	29.4		90	89.1	11	10.9	
Total	305	86.2	49	13.8		231	65.3	123	34.7		315	89.0	39	11.0		243	78.6	66	21.4		198	72.3	76	27.7		298	84.7	54	15.3	
**Family history of hypertension**																														
No	178	85.2	31	14.8	>0.05	138	66.3	70	33.7	>0.05	180	86.1	29	13.9	>0.05	151	82.5	32	17.5	>0.05	123	77.8	35	22.2	>0.05	182	87.9	25	12.1	>0.05
Yes	176	87.6	25	12.4		128	63.4	74	36.6		181	90.0	20	10.0		131	75.3	43	24.7		110	68.8	50	31.3		169	84.1	32	15.9	
Total	354	86.3	56	13.7		266	64.9	144	35.1		361	88.0	49	12.0		282	79.0	75	21.0		233	73.3	85	26.7		351	86.0	57	14.0	

Association assessed by Pearson’s Chi-squared test.

**Table 5:** Association of salt-related behavioral practices with knowledge, attitude, socio-demographic characteristics, and blood pressure (multiple regression analysis).

	Check Food Labels for the Salt/Sodium Content			Avoid Eating Packaged, Ready-to-Eat Foods			Use Spices/Herbs Instead of Salt When Cooking			Avoid Eating Food from Fast Food Restaurants			Purchase Foods Labelled “No Added Salt”, “Salt Reduced” or “Reduced Sodium”			When Eating out, Ask to Have Meals Prepared Without Salt			How Often Are Salt Shakers Placed on Your Table			Place Salt Shakers on Table			Consume Processed Food Products with High Salt Content			Reducing Salt Intake is Important to Me		
Variables	Cox and Snell R^2^ = 46.1%; Nagelkerke R^2^ = 69			Cox and Snell R^2^ = 16.2%; Nagelkerke R^2^ = 22.1			Cox and Snell R^2^ = 11.3%; Nagelkerke R^2^ = 15			Cox and Snell R^2^ = 22.9%; Nagelkerke R^2^ = 31.6			Cox and Snell R^2^ = 17.9%; Nagelkerke R^2^ = 26.2			Cox and Snell R^2^ = 13%; Nagelkerke R^2^ = 22.9			Cox and Snell R^2^ = 20.6%; Nagelkerke R^2^ = 33.6			Cox and Snell R^2^ = 22.6%; Nagelkerke R^2^ = 30.9			Cox and Snell R^2^ = 24.9%; Nagelkerke R^2^ = 50.5			Cox and Snell R^2^ = 19%; Nagelkerke R^2^ = 34.4		
	O.R.	β	[95% CI]	O.R.	β	[95% CI]	O.R.	β	[95% CI]	O.R.	β	[95% CI]	O.R.	β	[95% CI]	O.R.	β	[95% CI]	O.R.	β	[95% CI]	O.R.	β	[95% CI]	O.R.	β	[95% CI]	O.R.	β	[95% CI]
**Gender**																														
Male	1		(ref)	1		(ref)	1		(ref)	1		(ref)	1		(ref)	1		(ref)	1		(ref)	1		(ref)	1			1		(ref)
Female	0.77	−0.26	(0.06–9.34)	3.85	1.35	(1.07–13.82)	0.96	−0.04	(0.29–3.14)	2.18	0.78	(0.59–8.04)	0.11	−2.21	(0.02–0.60)	1.65	0.5	(0.22–12.15)	0.99	−0.01	(0.17–5.71)	1.7	0.53	(0.42–6.89)	3.22	1.17		0.12	−2.12	(0.01–0.99)
**Education**																														
Secondary school and below	1		(ref)	1		(ref)	1		(ref)	1		(ref)	1		(ref)	1		(ref)	1		(ref)	1		(ref)	1		(ref)	1		(ref)
High school	0.02	−3.91	(0.00–1.13)	8.25	2.11	(1.02–66.86)	0.99	−0.01	(0.17–5.93)	19.95	2.99	(1.81–219.46)	0.5	−0.69	(0.05–5.59)	2.17	0.77	(0.14–34.74)	3.24	1.18	(0.18–57.55)	9.59	2.26	(1.12–83.15)	5.99	1.79	(0.18–195.42)	0.07	−2.66	(0.02–2.22)
University and postgraduation	0.04	−3.22	(0.00–2.63)	5.89	1.77	(0.86–40.16)	0.53	−0.63	(0.10–2.89)	11.87	2.47	(1.36–103.56)	0.34	−1.08	(0.04–3.36)	2.89	1.06	(0.24–35.53)	1.96	0.67	(0.15–25.7)	5.69	1.74	(0.78–41.76)	1.61	0.48	(0.10–24.79)	0.07	−2.66	(0.00–1.83)
**Blood pressure**																														
Normotensive	1		(ref)	1		(ref)	1		(ref)	1		(ref)	1		(ref)	1		(ref)	1		(ref)	1		(ref)	1		(ref)	1		(ref)
Hypertensive	6.65	1.89	(0.68–64.95)	0.55	−0.6	(0.15–1.90)	1.19	0.17	(0.37–3.86)	0.61	−0.49	(0.15–2.44)	0.39	−0.94	(0.09–1.81)	0.61	−0.49	(0.12–3.22)	0.59	−0.53	(0.11–3.38)	0.85	−0.16	(0.22–3.29)	0.53	−0.63	(0.06–5.25)	0.24	−1.43	(0.04–1.58)
**Family history of hypertension**																														
No	1		(ref)	1		(ref)	1		(ref)	1		(ref)	1		(ref)	1		(ref)	1		(ref)	1		(ref)	1		(ref)	1		(ref)
Yes	1.48	0.39	(0.29–7.38)	0.69	−0.37	(0.292–1.63)	2.15	0.77	(0.96–4.81)	1.28	0.25	(0.51–3.21)	0.67	−0.4	(0.24–1.88)	0.56	−0.58	(0.15–2.02)	0.42	−0.87	(0.12–1.46)	0.44	−0.82	(0.17–1.14)	0.16	−1.83	(0.08–1.59)	0.92	−0.08	(0.25–3.34)
**Age**	1.05	0.05	(0.97–1.15)	1.06	0.06	(1.002–1.121)	1.04	0.04	(0.99–1.09)	1.1	0.1	(1.03–1.19)	0.99	−0.01	(0.93–1.06)	1.07	0.07	(1.00–1.14)	1.02	0.02	(0.95–1.08)	1.01	0.01	(0.96–1.07)	1.04	0.04	(0.96–1.13)	0.96	−0.04	(0.87–1.05)
**Knowledge score**	0.42	−0.87	(0.26–0.69)	1.15	0.14	(0.939–1.413)	1.07	0.07	(0.88–1.30)	1.23	0.21	(0.98–1.55)	0.84	−0.17	(0.65–1.08)	1.17	0.16	(0.89–1.52)	1.16	0.15	(0.88–1.51)	0.89	−0.12	(0.73–1.09)	0.63	−0.46	(0.39–1.01)	1.39	0.33	(1.01–1.94)
**Attitude score**	0.64	−0.45	(0.31–1.32)	1.74	0.55	(1.187–2.565)	1.14	0.13	(0.81–1.63)	1.78	0.58	(1.17–2.68)	1.03	0.03	(0.63–1.69)	1.96	0.67	(1.09–3.51)	3.87	1.35	(1.98–7.57)	2.82	1.04	(1.77–4.48)	7.61	2.03	2.46–23.59	2.68	0.99	(1.37–5.25)

Dependent variables: “Check food lables for the salt/sodium content”, “Avoided eating packaged, ready-to-eat foods”, “Used spices/herbs instead of salt when cooking”, “Avoid eating food from fast food restaurants”, “Purchase foods labeled ‘no added salt’, ‘salt reduced’ or ‘reduced sodium’”, “When eating out, ask to have your meal prepared without salt”, “How Often Are Salt Shakers Placed on Your Table”, “Place salt shakers on table”, “Consume processed food products with a high salt content”, and “Reducing salt intake important to me”. Independent variables: gender, education, blood pressure, family history of hypertension, age, knowledge score, and attitude score.

## Data Availability

The data presented in this study are available upon reasonable request from the corresponding author.

## Supplementary Materials

The following supporting information can be downloaded at https://www.mdpi.com/article/10.3390/foods14010141/s1, Table S1: Behavioral practices to reduce salt intake performed in the past month by gender and blood pressure characteristics; Table S2: Association of salt-related behaviors with knowledge; File S1: Knowledge, attitudes, and behaviors related to dietary salt intake questionnaire; File S2: Validity and reliability analyses of the knowledge questions regarding salt and salt consumption.
