# Supplementary material for: Dietary Salt-Related Knowledge, Attitudes and Behaviors in Healthy and Hypertensive Turkish Adults from Food Choice Perspective
Source: Foods. 2025 Jan 6;14(1):141. doi: 10.3390/foods14010141 (PMC11720551; doi:10.3390/foods14010141)
Supplement: Supplementary file 1 [file foods-14-00141-s001.zip › File S1.pdf]

Knowledge, Attitudes and Behaviours related to Dietary Salt Intake Questionnaire

The following questions ask about your knowledge, attitudes and behaviour towards dietary salt. Please answer each question by selecting the most suitable option. Thank you for taking the time to complete this survey.

1.DEMOGRAPHIC INFORMATION

**Name, Surname**:		
**What is your sex?** [1]	**1**.Male	**2.** Female
**What is your age in complete years?** [1]		
**What is the highest level of education and training you have completed?**[1]	1. Literate	4. High school
	2. Primary school	5. University
	3. Secondary school	6. Postgraduation
**Who do you live with?**	**1.** Alone	**2.** With family
	**3.** With friend	**4.** Other (please specify..)

2.GENERAL INFORMATION

**Do you have hypertension patients in your family (mother, father, sibling, etc.)?**[1]			
**1.** No	**2.** Yes		
**Have you ever been diagnosed with or suffered from one or more of the following conditions? (You may select more than one)** [1]			
**1.** High blood pressure	**2**. Heart attack	**3**. Renal calculus	**7.** Stroke
**4.** Asthma	**5.** Osteoporosis	**6**. Gastric cancer	**8.** Heart disease
**Do you smoke?**			
**1.** No	**2.** Yes		
**If yes to high blood pressure response, do you currently take medication for the control of your blood pressure?**[1]			
**1.** No	**2.**Yes (Please specify….)		

Have you ever received any advice from your doctor or a health professional to reduce your intake of salt/sodium and/or salty foods?[1,17]

1. No **2.** Yes

Are you the main person who does the grocery shopping in your household?[1,3]

1. No **2.** Yes

Do you have children under 18 or are you responsible for caring for a child under 18?[1]

1. No **2.** Yes

3. VIEWS ON DİETARY SALT AND SALT INTAKE

*Knowledge towards dietary salt**

Do you think that eating too much salt could damage your health? [1,2,4,8,10,11,13,15,16,18]

1. No **2. Yes** **3.** Don't know

On some food products information about the amount of sodium within a food product is displayed on the food label. What is the relationship between salt and sodium? [1,3,5,6]

1. They are the same **2. Salt contains sodium** **3.** Sodium contains salt **4.** Don't know

How much salt do you think Turkish society eats? [1]

1. Too much **2.** Much **3.** The right amount **4.** Little **5.** Too little **6.** Don't know

Which of the following do you think is the main source of salt in the in the diet of Turkish population diet? [1,4,5,6]

1. Salt added during cooking

2. Salt taken with foods such as olives, cheese, tomato paste and pickles

3. Salt intake with bread consumption

4. Salt used in the table

Himalayan salt, pink salt, sea salt and gourmet salts are healthier than regular table salt.[1]

1. No **2.** Yes **3.** Don't know

Health professionals recommend that we should eat no more than a certain amount of salt each day. How much salt do you think this is? [1,2,4,5,6,7,9,14,16,17,18]

1. 3 grams (about ½ a teaspoon) **2. 5 grams (about 1 teaspoon)** **3.** 8 grams (about 1 teaspoons)

4. 10 grams (about 2 teaspoons) **5.** 15 grams (about 3 teaspoons) **6.** Don't know

Do you have any information about the "Reduction of Excessive Salt Consumption Program in Turkey" of the Ministry of Health of the Republic of Turkey? [1,5]

1. No **2. Yes** **3.** Don't know

*(Correct answers are in bold for each question.)

Which, if any, of the following conditions do you think is linked to eating too much salt? [1,3,4,6,14,16,17,18]

Health problem	Yes	No	Don't know/Not sure
Hypertension			
Kidney diseases			
Heart disease<br>Heart attack			
Stroke			
Stomach cancer			
Osteoporosis			

Attitudes and behaviors toward dietary salt

How often are salt shakers placed on your table? [1,6,8,11,12,13]

1. Usually **2.** Sometimes **3.** Never

How often do you add salt before tasting or while eating? [1,2,6,8,10,11,12,13,15]

1. Usually **2.** Sometimes **3.** Never

How often is salt added during cooking?[1,2,6,8,10,11,12,15]

1. Usually **2.**Sometimes

How often do you consume processed food products with a high salt content? [1,2,8,10,11,15]

1. Usually **2.** Sometimes **3.** Never

Are you trying to cut down on the amount of salt you eat? [1,6,8]

1. No **2.** Yes **3.** Don't know

Is reducing salt intake important to you? [1,2,4,6,7,10,11,16]

1. No **2.** Yes **3.** Don't know

Behaviors towards dietary salt

Below are some common ways that can help reduce salt consumption. Indicate your practices related to these in the last 1 month by ticking the boxes with a cross (X).[1,2,3,6,7,11,18]

Behaviours	Never do this	Sometimes do this	Often do this
Looked at a food label to check the salt/sodium content of a food item			
Avoided eating packaged, ready-to-eat foods			
Used spices/herbs instead of salt when cooking			
Avoided eating food from fast food restaurants			
Avoided eating food from an Asian style restaurant or takeaway store (e.g. Chinese, Thai, Indian)			
Purchased foods labelled “no added salt”, “salt reduced” or “reduced sodium”			
When eating out, asked to have your meal prepared without salt			

References

1. Grimes CA, Kelley SJ, Stanley S, et al. Knowledge, attitudes and behaviours related to dietary salt among adults in the state of Victoria, Australia 2015. BMC Public Health. 2017;17(1):532. Published 2017 May 30. doi:10.1186/s12889-017-4451-0

2. Sparks E, Paterson K, Santos JA, Trieu K, Hinge N, Tarivonda L, Snowdon W, Johnson C, Webster J. Salt-Related Knowledge, Attitudes, and Behaviors on Efate Island, Vanuatu. Int J Environ Res Public Health. 2019 Mar 21;16(6):1027. doi: 10.3390/ijerph16061027.

3. Westrick SC, Garza KB, Stevenson TL, Oliver WD. Association of blood pressure with sodium-related knowledge and behaviors in adults with hypertension. J Am Pharm Assoc (2003). 2014 Mar-Apr;54(2):154-8. doi: 10.1331/JAPhA.2014.13173.

4. Chen S, Shan LC, Tao W, Lu T, Regan Á, Han H, Guo L, Deng T, Wall P. A survey of Chinese consumers' knowledge, beliefs and behavioural intentions regarding salt intake and salt reduction. Public Health Nutr. 2020 Jun;23(8):1450-1459. doi: 10.1017/S1368980019003689.

5. Regan Á, Shan CL, Wall P, McConnon Á. Perspectives of the public on reducing population salt intake in Ireland. Public Health Nutr. 2016;19(7):1327-1335. doi:10.1017/S1368980015002530

6. Nasreddine L, Akl C, Al-Shaar L, Almedawar MM, Isma'eel H. Consumer knowledge, attitudes and salt-related behavior in the middle-east: the case of Lebanon. Nutrients. 2014;6(11):5079–102.

7. Iaccarino Idelson P, D'Elia L, Cairella G, Sabino P, Scalfi L, Fabbri A, Galletti F, Garbagnati F, Lionetti L, Paolella G, Simonetti P, Strazzullo P, On Behalf Of The Sinu-Gircsi Working Group. Salt and Health: Survey on Knowledge and Salt Intake Related Behaviour in Italy. Nutrients. 2020 Jan 21;12(2):279. doi: 10.3390/nu12020279.
8. Leyvraz M, Mizéhoun-Adissoda C, Houinato D, et al. Food Consumption, Knowledge, Attitudes, and Practices Related to Salt in Urban Areas in Five Sub-Saharan African Countries. Nutrients. 2018;10(8):1028. Published 2018 Aug 7. doi:10.3390/nu10081028

9. Du X, Fang L, Xu J, et al. The association of knowledge, attitudes and behaviors related to salt with 24-h urinary sodium, potassium excretion and hypertensive status. Sci Rep. 2022;12(1):13901. Published 2022 Aug 16. doi:10.1038/s41598-022-18087-x

10. Ghimire K, Adhikari TB, Rijal A, Kallestrup P, Henry ME, Neupane D. Knowledge, attitudes, and practices related to salt consumption in Nepal: Findings from the community-based management of non-communicable diseases project in Nepal (COBIN). J Clin Hypertens (Greenwich). 2019;21(6):739-748.

11. Bhattacharya, Sudip1,; Thakur, J. S.1; Singh, Amarjeet1. Knowledge attitude, and practice regarding dietary salt intake among urban slum population of North India. Journal of Family Medicine and Primary Care 7(3):p 526-530, May–Jun 2018. | DOI: 10.4103/jfmpc.jfmpc_60_17

12. Alawwa I, Dagash R, Saleh A, Ahmad A. Dietary salt consumption and the knowledge, attitudes and behavior of healthy adults: a cross-sectional study from Jordan. Libyan J Med. 2018;13(1):1479602. doi:10.1080/19932820.2018.1479602

13. D'Elia L, Brajović M, Klisic A, et al. Sodium and Potassium Intake, Knowledge Attitudes and Behaviour Towards Salt Consumption Amongst Adults in Podgorica, Montenegro. Nutrients. 2019;11(1):160. Published 2019 Jan 13. doi:10.3390/nu11010160

14. Fan F, Li Y, Li L, et al. Salt-Related Knowledge, Attitudes, and Behaviors and Their Relationship with 24-Hour Urinary Sodium Excretion in Chinese Adults. Nutrients. 2022;14(20):4404. Published 2022 Oct 20. doi:10.3390/nu14204404

15. Al-Mawali A, D'Elia L, Jayapal SK, et al. National survey to estimate sodium and potassium intake and knowledge attitudes and behaviours towards salt consumption of adults in the Sultanate of Oman. BMJ Open. 2020;10(10):e037012. Published 2020 Oct 23. doi:10.1136/bmjopen-2020-037012

16. Aparna P, Salve HR, Anand K, Ramakrishnan L, Gupta SK, Nongkynrih B. Knowledge and behaviors related to dietary salt and sources of dietary sodium in north India. J Family Med Prim Care. 2019;8(3):846-852. doi:10.4103/jfmpc.jfmpc_49_19

17. Qin Y, Li T, Lou P, et al. Salt intake, knowledge of salt intake, and blood pressure control in Chinese hypertensive patients. J Am Soc Hypertens. 2014;8(12):909-914. doi:10.1016/j.jash.2014.09.018

18. Land MA, Webster J, Christoforou A, et al. The association of knowledge, attitudes and behaviours related to salt with 24-hour urinary sodium excretion. Int J Behav Nutr Phys Act. 2014;11(1):47. Published 2014 Apr 4. doi:10.1186/1479-5868-11-47
