# Supplementary material for: Dietary Salt-Related Knowledge, Attitudes and Behaviors in Healthy and Hypertensive Turkish Adults from Food Choice Perspective
Source: Foods. 2025 Jan 6;14(1):141. doi: 10.3390/foods14010141 (PMC11720551; doi:10.3390/foods14010141)
Supplement: Supplementary file 1 [file foods-14-00141-s001.zip › File S2.pdf]

The Discriminative Item Analysis of Multiple-Choice Knowledge Questions

Item difficulty index and item discrimination index results for the items

Items	Difficulty index	Discrimination index	KR-20
Do you think that eating too much salt could damage your health?	0.90	0.24	
What is the relationship between salt and sodium?	0.30	0.32	
How much salt do you think Turkish society eats?	0.80	0.34	
Which of the following do you think is the main source of salt in the in the diet of Turkish population diet?	0.54	0.32	
Himalayan salt, pink salt, sea salt and gourmet salts are healthier than regular table salt.	0.18	0.37	
Health professionals recommend that we should eat no more than a certain amount of salt each day. How much salt do you think this is?	0.40	0.26	
Do you have any information about the "Reduction of Excessive Salt Consumption Program?	0.30	0.52	0.712
Do you think it is linked to eating too much salt with hypertension ?	0.06	0.51	
Do you think it is linked to eating too much salt with kidney diseases?	0.04	0.58	
Do you think it is linked to eating too much salt with heart disease?	0.66	0.59	
Do you think it is linked to eating too much salt with stroke?	0.10	0.58	
Do you think it is linked to eating too much salt with stomach cancer?	0.28	0.60	
Do you think it is linked to eating too much salt with osteoporosis?	0.24	0.64	
Is reducing salt intake important to you?	0.30	0.43	

The literature states that a knowledge survey should include all items with difficulty, ease, and medium difficulty according to the difficulty index [1]. Moreover, items with an item difficulty index of 0.29 and below are classified as difficult, those between 0.30 and 0.49 are classified as medium difficulty, and those above 0.50 are classified as easy [1]. Accordingly, our questionnaire contains difficult, easy and medium-difficulty items. According to item discrimination index, items 0.40 and above are considered as very good items, 0.30-0.39 are quite good, 0.20-0.29 can be corrected and can still be improved, and 0.19 and below are considered as very weak and must be removed [1]. All items are valid and do not need to be removed.

Moreover, This knowledge questionnaire's internal consistency reliability was evaluated with The Kuder-Richardson formula-20 (KR20) and and moderate reliability was found [2]. As a result, the knowledge questionnaire was valid and reliable.

References

1.) Hasançebi, B., Terzi, Y., & Küçük, Z. (2020). Distractor Analysis Based on Item Difficulty Index and Item Discrimination Index. Gümüşhane Üniversitesi Fen Bilimleri Dergisi, 10(1), 224-240.

2.) Salvucci, S., Walter, E., Conley, V., Fink, S., & Saba, M. (1997). Measurement error studies at the National Center for Education Statistics (NCES). Washington D. C.: U. S. Department of Education.
