# Supplementary material for: Dietary Salt-Related Knowledge, Attitudes and Behaviors in Healthy and Hypertensive Turkish Adults from Food Choice Perspective
Source: Foods. 2025 Jan 6;14(1):141. doi: 10.3390/foods14010141 (PMC11720551; doi:10.3390/foods14010141)
Supplement: Supplementary file 1 [file foods-14-00141-s001.zip › Tables S1 and S2.pdf]

Table S1. Behavioral practices to reduce salt intake performed in the past month by blood pressure and gender characteristics

Behavioral practices	Total		Normotensive		Hypertensive		p	Male		Female		p
	n	%	n	%	n	%		n	%	n	%	
**Looked at a food label to check the salt/sodium content of a food item**												
Never do this	225	72.8	176	73.3	49	71.0		118	74.7	171	70.7	
Sometimes do this	58	18.8	46	19.2	12	17.4	>0.05	28	17.7	47	19.4	>0.05
Often do this	26	8.4	18	7.5	8	11.6		12	7.6	24	9.9	
**Avoided eating packaged, ready-to-eat foods**												
Never do this	125	40.5	94	39.2	31	44.9		68	43.3	87	36.3	
Sometimes do this	87	28.2	72	30.0	15	21.8	>0.05	41	26.1	70	29.2	>0.05
Often do this	97	31.4	74	30.8	23	33.3		48	30.6	83	34.6	
**Used spices/herbs instead of salt when cooking**												
Never do this	155	50.2	120	50.0	35	50.7		83	53.2	120	50.2	
Sometimes do this	74	23.9	62	25.8	12	17.4	>0.05	33	21.2	54	22.6	>0.05
Often do this	80	25.9	58	24.2	22	31.9		40	25.6	65	27.2	
**Avoided eating food from fast food restaurants**												
Never do this	121	39.2	98	40.8	23	33.3		69	44.2	87	36.3	
Sometimes do this	69	22.3	59	24.6	10	14.5	<0.05	29	18.6	64	26.7	>0.05
Often do this	119	38.5	83	34.6	36	52.2		58	37.2	89	37.1	
**Avoided eating food from an Asian style restaurant or takeaway store (e.g. Chinese, Thai, Indian)**												
Never do this	149	37.8	114	37.9	35	37.6		77	49.3	132	55.6	
Sometimes do this	39	9.4	31	10.6	8	8.6	>0.05	13	8.3	26	11.0	>0.05
Often do this	145	36.8	110	36.5	35	37.6		66	42.3	79	33.3	
**Purchased foods labelled "no added salt", "salt reduced" or "reduced sodium"**												
Never do this	190	61.5	152	63.3	38	55.1	>0.05	107	68.6	140	59.3	>0.05

Sometimes do this	71	23.0	56	23.3	15	21.7		23	14.7	62	26.3	
Often do this	48	15.5	32	13.3	16	23.2		26	16.7	34	14.4	
**When eating out, asked to have your meal prepared without salt**												
Never do this	246	79.6	193	80.4	53	76.8		115	74.7	203	85.3	
Sometimes do this	39	12.6	33	13.8	6	8.7	<0.05	24	15.6	20	8.4	<0.05
Often do this	24	7.8	14	5.8	10	14.5		15	9.7	15	6.3	

Association assessed by Pearson's Chi-squared test

Table S2. Association of salt-related behaviors with knowledge

Knowledge about dietary salt	How often are salt shakers placed on your table?					How often do you add salt before tasting or while eating?					How often do you consume processed food products with a high salt content?					Looked at a food label to check the salt/sodium content of a food item					Purchased foods labelled “no added salt”, “salt reduced” or “reduced sodium”					Are you trying to cut down on the amount of salt you eat?				
	Usually		Never		p	Usually		Never		p	Usually		Never		p	Usually		Never		p	Usually		Never		p	Yes		No		p
	n	%	n	%		n	%	n	%		n	%	n	%		n	%	n	%		n	%	n	%		n	%	n	%	
**What is the relationship between salt and sodium?**																														
True	179	85.2	31	14.8	>0.05	132	62.9	78	37.1	>0.05	189	0.9	21	0.1	>0.05	144	77.4	42	22.6	>0.05	128	72.7	48	27.3	>0.05	185	88.5	24	11.5	>0.05
False	60	92.3	5	7.7		45	68.2	21	31.8		58	0.9	8	0.1		43	78.2	12	21.8		33	70.2	14	29.8		51	78.5	14	21.5	
Don’t know	107	85.6	18	14.4		83	66.9	41	33.1		105	0.8	20	0.2		90	84.1	17	15.9		70	76.9	21	23.1		106	85.5	18	14.5	
Total	346	86.5	54	13.5		260	65.0	140	35.0		352	0.9	49	0.1		277	0.4	71	20.4		231	73.6	83	26.4		342	85.9	56	14.1	
**How much salt do you think Turkish society eats?**																														
True	209	87.8	29	12.2	>0.05	159	0.7	80	0.3	>0.05	216	0.9	23	0.1	>0.05	168	78.5	46	21.5	>0.05	139	72.4	53	27.6	>0.05	199	83.6	39	16.4	>0.05
False	115	83.9	22	16.1		85	0.6	51	0.4		117	0.9	19	0.1		89	78,.1	25	21.9		72	72.7	27	27.3		123	90.4	13	9.6	
Don’t know	30	88.2	4	11.8		23	0.7	11	0.3		27	0.8	7	0.2		26	86.7	4	21.9		23	85.2	4	14.8		27	81.8	6	18.2	
Total	354	86.6	55	13.4		267	0.7	142	0.3		360	0.9	49	0.1		283	79.1	75	20.9		234	73.6	84	26.4		349	85.7	58	14.3	
**Which of the following do you think is the main source of salt in the in the diet of Turkish population diet?**																														
True	332	86.9	50	13.1	>0.05	252	0.7	130	0.3	>0.05	-	-	-	-	>0.05	15	68.2	7	31.8	>0.05	12	70.6	5	29.4	>0.05	-	-	-	-	>0.05
False	17	73.9	6	26.1		12	0.5	11	0.5		335	0.9	47	0.1		266	79.9	67	20.1		219	73.5	79	26.5		328	86.3	52	13.7	
Total	349	86.2	56	13.8		264	0.7	141	0.3		357	0.8	48	4.1		281	79.2	74	20.8		-	- -	-	-		346	85.9	57	14.1	
**Health professionals recommend that we should eat no more than a certain amount of salt each day. How much salt do you think this is?**																														
True	128	84.8	23	15.2	>0.05	102	0.7	50	0.3	>0.05	139	0.9	13	0.1	>0.05	106	78.5	29	21.5	>0.05	95	76.0	30	24.0	>0.05	129	85.4	22	14.6	>0.05
False	139	89.1	17	10.9		102	0.7	53	0.3		139	0.9	17	0.1		104	78.2	29	21.8		80	70,2	34	29.8		134	86.5	21	13.5	
Don’t know	82	85.4	14	14.6		58	0.6	38	0.4		81	0.8	15	0.2		70	82.4	15	17.6		57	75.0	19	25.0		80	84.2	15	15.8	
Total	349	86.6	54	13.4		262	0.7	141	0.3		359	0.9	45	0.1		280	79.3	73	20.7		232	73.7	83	26.3		343	85.5	58	14.5	
**Do you think hypertension is linked to eating too much salt?**																														
True	327	85.8	54	14.2	>0.05	243	0.6	138	0.4	>0.05	340	0.9	41	0.1	<0.05	267	79.7	68	20.3	>0.05	218	73.6	78	26.4	>0.05	327	86.3	52	13.7	>0.05
False	15	100	-	-		12	0.8	3	0.2		10	0.7	5	0.3		8	66.7	4	33.3		10	71.4	4	28.6		11	73.3	4	26.7	
Don’t know	9	81.8	2	18.2		9	0.8	2	0.2		9	0.8	2	0.2		6	66.7	3	33.3		5	62.5	3	37.5		10	90.9	1	9.1	
Total	351	86.2	56	13.8		264	0.6	143	0.4		359	0.9	48	0.1		281	78.9	75	21.1		233	73.3	85	26.7		348	85.9	57	14.1	
**Do you think stroke is linked to eating too much salt?**																														
True	202	82.8	42	17.2	>0.05	147	0.6	96	0.4	<0.05	215	0.9	28	0.1	>0.05	165	77.8	47	22.2	>0.05	135	71.4	54	28.6	>0.05	213	87.7	30	12.3	>0.05
False	51	92.7	4	7.3		41	0.7	14	0.3		47	0.9	8	0.1		38	84.4	7	15.6		33	76.7	10	23.3		45	81.8	10	18.2	
Don’t know	86	89.6	10	10.4		72	0.7	25	0.3		87	0.9	10	0.1		71	80.7	17	19.3		59	74.7	20	25.3		80	83.3	16	16.7	
Total	339	85.8	56	14.2		260	0.7	135	0.3		349	0.9	46	0.1		274	79.4	71	20.6		227	73.0	84	27.0		338	85.8	56	14.2	
**Do you think kidney diseases are linked to eating too much salt?**																														
True	309	85.1	54	14.9	>0.05	233	0.6	130	0.4	>0.05	327	0.9	36	0.1	<0.05	255	79.9	64	20.1	>0.05	207	72.6	78	27.4	>0.05	311	86.1	50	13.9	>0.05
False	20	100.0	0	0		14	0.7	6	0.3		14	0.7	6	0.3		11	78.6	3	21.4		12	80.0	3	20.0		15	7.0	5	25.0	

Don't know	18	90.0	2	10.0		15	0.8	5	0.3		16	0.8	4	0.2		13	72.2	5	27.8		13	81.3	3	18.8		18	90.0	2	10.0	
Total	347	86.1	56	13.9		262	0.7	141	0.3		357	0.9	46	0.1		279	79.5	72	20.5		232	73.4	84	26.6		344	85.8	57	14.2	
**Do you think heart diseases are linked to eating too much salt?**																														
True	261	84.5	48	15.5	>0.05	198	64.3	110	35.7	>0.05	274	89.0	34	11.0	>0.05	212	78.5	58	21.5	>0.05	173	71.8	68	28.2	>0.05	268	87.0	40	13.0	>0.05
False	33	97.1	1	2.9		25	73.5	9	26.5		30	88.2	4	11.8		21	77.8	6	22.2		19	76.0	6	24.0		27	79.4	7	20.6	
Don't know	48	87.3	7	12.7		37	66.1	19	33.9		48	85.7	8	14.3		43	84.3	8	15.7		37	78.7	10	21.3		45	81.8	10	18.2	
Total	342	85.9	56	14.1		260	65.3	138	34.7		352	88.4	46	11.6		276	79.3	72	20.7		229	73.2	84	26.8		340	85.6	57	14.1	
**Do you think stomach cancer is linked to eating too much salt?**																														
True	123	86.0	20	14.0	>0.05	89	62.7	53	37.3	>0.05	130	90.9	13	9.1	>0.05	98	77.8	28	22.2	>0.05	83	70.9	34	29.1	>0.05	126	88.1	17	11.9	>0.05
False	73	85.8	12	14.1		63	74.1	22	25.9		76	89.4	9	10.6		56	82.4	12	17.6		52	75.4	17	24.6		69	81.2	16	18.8	
Don't know	140	85.4	24	14.6		105	63.6	60	36.4		142	86.1	23	13.9		119	79.9	30	20.1		92	73.6	33	26.4		140	85.9	23	14.1	
Total	336	85.7	56	14.3		257	65.6	135	34.4		348	88.5	45	11.5		273	79.6	70	20.4		227	73.0	84	27.0		335	85.7	56	14.3	
**Do you think osteoporosis is linked to eating too much salt?**																														
True	115	87.1	17	12.9	>0.05	86	65.6	45	34.4	>0.05	117	88.6	15	11.4	>0.05	88	77.9	25	22.1	>0.05	72	69.2	32	30.8	>0.05	116	88.5	15	11.5	>0.05
False	66	88.0	9	12.0		55	73.3	20	26.7		66	8.08	9	12.0		47	79.7	12	20.3		41	70.7	17	29.3		61	81.3	14	18.7	
Don't know	156	83.9	30	16.1		117	62.6	70	37.4		165	88.2	22	11.8		138	80.7	33	19.3		114	76.5	35	23.5		160	86.0	26	14.0	
Total	337	85.8	56	14.2		258	65.6	135	34.4		348	88.3	46	11.7		273	79.6	70	20.4		227	73.0	84	27.0		337	86.0	55	14.0	

Association assessed by Pearson's Chi-squared test
